# Supplementary material for: In-Vitro Selection of Ceftazidime/Avibactam Resistance in OXA-48-Like-Expressing Klebsiella pneumoniae: In-Vitro and In-Vivo Fitness, Genetic Basis and Activities of β-Lactam Plus Novel β-Lactamase Inhibitor or β-Lactam Enhancer Combinations
Source: Antibiotics (Basel). 2021 Oct 29;10(11):1318. doi: 10.3390/antibiotics10111318 (PMC8614831; doi:10.3390/antibiotics10111318)
Supplement: Supplementary file 1 [file antibiotics-10-01318-s001.zip › antibiotics-1401955-supplementary.pdf]

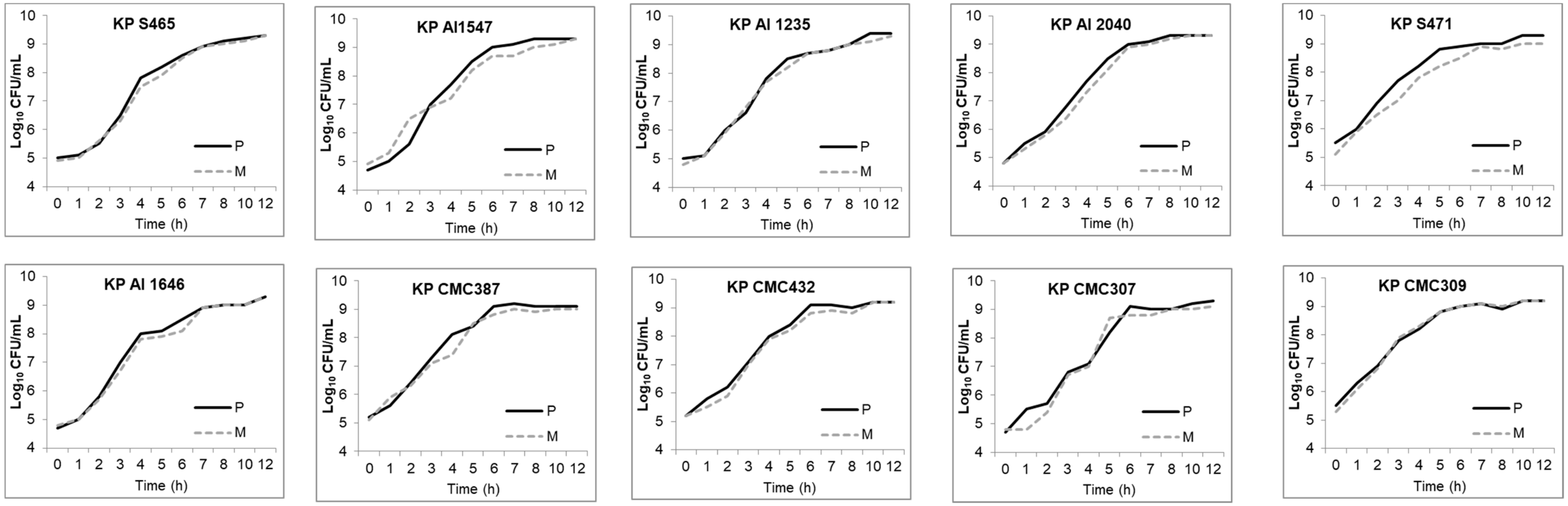

Figure S1. Comparative growth kinetics of terminal mutants and their respective parent strains of *bla*$_{\text{OXA-48-like}}$ -harbouring *K. pneumoniae* in CAMHB medium.

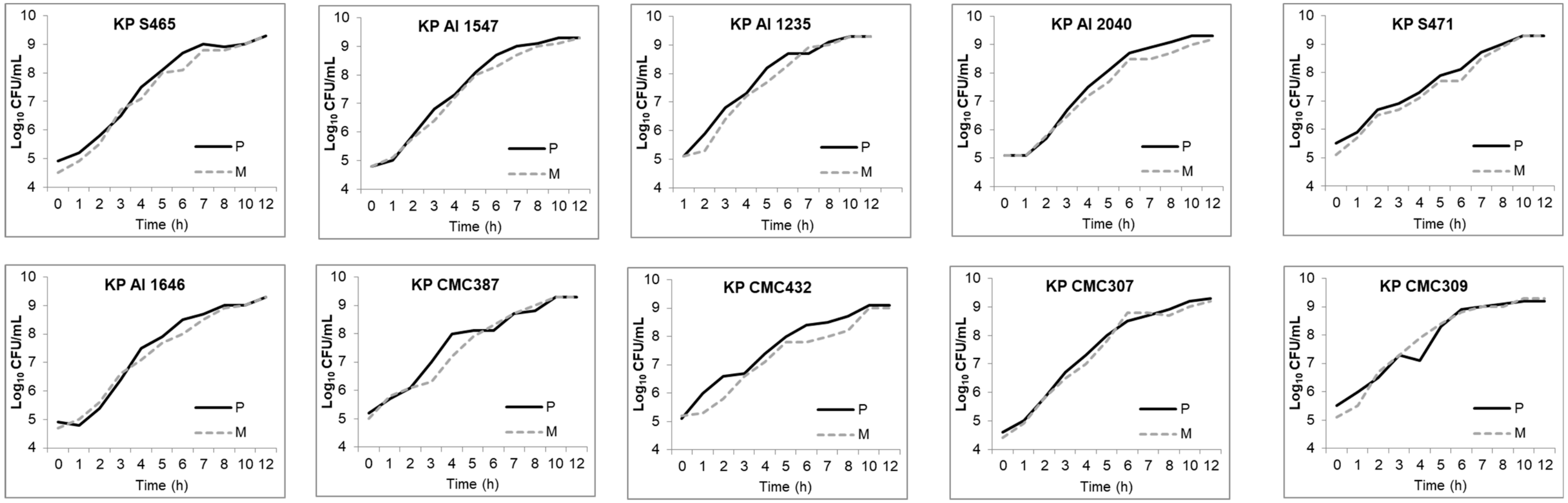

Figure S2. Comparative growth kinetics of terminal mutants and their respective parent strains of *bla*$_{\text{OXA-48-like}}$ -harbouring *K. pneumoniae* in M9 minimal medium**.**
